# Murici (*Byrsonima crassifolia* (L.) *Kunth* and *verbascifolia* (L.)) and Tapereba (*Spondias mombin*) Improve Hepatic and Inflammatory Biomarkers in High-Fat-Diet Rats

**DOI:** 10.3390/foods12020255

**Published:** 2023-01-05

**Authors:** Vanessa Rosse de Souza, Thuane Passos Barbosa Lima, Teresa Palmiciano Bedê, Sabrina Baptista Alves Faria, Renata Alves, Alana Louzada, Bianca Portugal Tavares de Moraes, Adriana Ribeiro Silva, Cassiano Felippe Gonçalves de Albuquerque, Vilma Blondet de Azeredo, Anderson Junger Teodoro

**Affiliations:** 1 Food and Nutrition Program, Functional Foods Laboratory, Federal University of the State of Rio de Janeiro, Rio de Janeiro 22290-240, Brazil; 2 Department of Nutrition and Dietetics, Fluminense Federal University, Rio de Janeiro 24020-140, Brazil; 3 Membrane Transport Laboratory, State University of Rio de Janeiro, Rio de Janeiro 24020-140, Brazil; 4 Immunopharmacology Laboratory, Biomedical Institute, Federal University of the State of Rio de Janeiro, Rio de Janeiro 21941-902, Brazil; 5 Immunopharmacology Laboratory, Oswaldo Cruz, Oswaldo Cruz Foundation (FIOCRUZ), Rio de Janeiro 21040-900, Brazil

**Keywords:** anti-inflammatory activity, antioxidants, apoptosis, carotenoids, functional nutrition, high-fat diet, murici, tapereba

## Abstract

The present study investigated the effects of murici and tapereba on improving hepatic and inflammatory biomarkers in high-fat-diet rats. Female Wistar rats were divided into five groups (*n* = 10/group): control (CON), high-fat diet (HF), murici drink + high-fat diet (Mu-HF), tapereba drink + high-fat diet (Tap-HF), and murici and tapereba blend drink + high-fat diet (MT-HF). Drinks were offered daily for 60 days, following which body and liver weights, hepatosomatic indexes, serum parameters, inflammatory profile, and antioxidant activity (DPPH and ORAC) were analyzed. The cell death of hepatic cells was evaluated using flow cytometry. It was observed that weight gain was similar among the groups, while glycemia was lower in the MT-HF group. A high-fat diet increased the concentration of cholesterol total, ALT, IL-1β (in plasma and liver), and TNF-α (in the liver), and this was reduced by treatment with the fruit-based beverages. The other evaluated parameters showed no statistically significant difference. Compared to the CON and HF groups, the groups that received the drinks had higher cellular antioxidant activity and reduced oxidative stress, lipid oxidation, and development of pro-inflammatory cytokines, such as IL-1β. A high-fat diet induced higher cell death in hepatic tissue, which was prevented by the murici, tapereba, and the fruit-blend drinks. The consumption of murici, tapereba, and fruit-blend-based beverages showed beneficial effects on liver metabolism; therefore, they may serve as a nutritional approach for preventing and treating non-alcoholic liver disease.

## 1. Introduction

The Amazon region contains several native and exotic fruit species which have attracted interest owing to increasing awareness of their nutritional and therapeutic value [1,2]. The variety among these species is affected by the existing flora, migration, and evolution of new species [3]. Consumption of fruit-based beverages has increased on the local Brazilian and international market, which presents an opportunity for local producers to access markets where buyers value foods and nutrients with the potential for disease prevention or health promotion [4].

There is great concern among consumers worldwide about healthy eating habits, which is why there is an association of fruits as a primary source of nutrients and functional compounds [5]. From a public health perspective, it is vital to identify new plant foods which can prevent nutritional deficiencies and chronic diseases [6]. Amazonian fruits have considerable amounts of micronutrients and essential nutrients, such as mineral salts, fibers, vitamins, and phenolic compounds [4].

Poor habits, such as excessive fat consumption, are harmful to health. Obesity results from such habits and is considered a risk factor for several chronic diseases and metabolic disorders, including fatty liver disease, insulin resistance, and dyslipidemia. Thus, a high-fat diet plays a central role in the production of oxidant events such as hepatic steatosis and atherosclerosis [7,8]. Excessive consumption of fats generates an increased flow of lipids to the liver, promoting a state of lipotoxicity in the organ, associated with a high level of oxidative stress and reduction of the body’s antioxidant defense. The accumulation of pro-inflammatory cytokines, production of oxidative stress, and damage to specific biomolecules are often associated with the intake of saturated fats and fat accumulation in tissues. Additionally, obese individuals have higher tumoral necrosis factor-alpha (TNF-α), interleukin-1 beta (IL)-1β, and IL-6 levels, all of which are produced by macrophages [9].

There is an inverse link between the intake of carotenoid-containing foods and the risk of oxidative-stress-induced diseases [10]. They protect against diseases, mainly owing to their antioxidant effect, regulation of lipid enzyme activity, ability to induce adipocyte differentiation, and improvement of the plasma lipid profile in rats fed a high-fat diet [11].

It is relevant to study the effect of murici and tapereba fruit-based beverages consumption on the function and integrity of the hepatic tissue, since benefits of their bioactive compounds in the prevention of liver diseases and injuries are expected. This study aimed to evaluate the effect of murici and tapereba fruit-based beverages from the Amazon region on liver tissue changes in Wistar rats that were fed a high-fat diet.

## 2. Materials and Methods

### 2.1. Animals and Diet

The animals were housed and handled following the Animal Research: Reporting of In Vivo Experiments guidelines [12]. The Ethics Committee on Animal Use of the Fluminense Federal University approved all procedures by the Animal Ethics Committee from Federal Fluminense University (protocol under number 00216/10). Female Wistar rats, weighing 180 g on average, were maintained in individual standard cages of polypropylene, on a 12 h: 12 h light–dark cycle, with controlled temperature (24 ± 2 °C) and relative moisture (60 ± 5%), with food and water provided ad libitum for 60 days.

**Figure 1:**
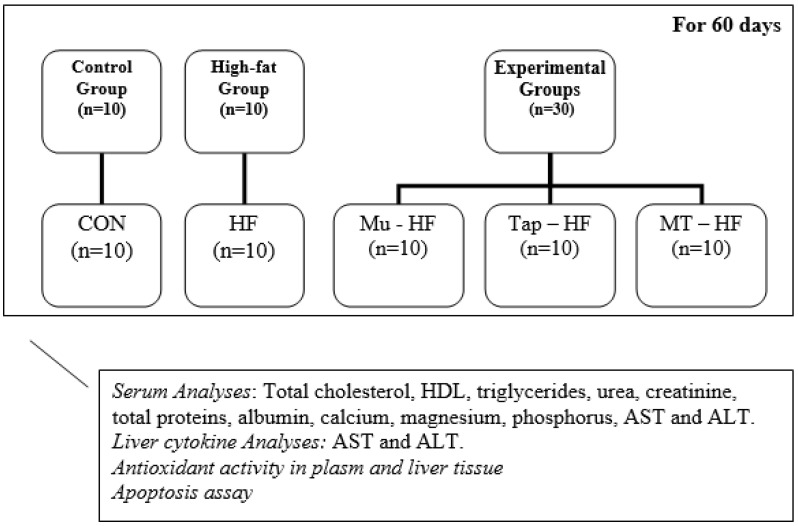
This empirical model: CON: control group. HF: high-fat group. Experimental Groups: Mu-HF: group that received murici fruit-based beverages and high-fat diet; Tap-HF: group that received tapereba fruit-based beverages and high-fat diet; MT-HF: group that received murici and tapereba blend fruit-based beverages and high-fat diet. HDL: high-density lipoprotein. AST: aspartate aminotransferase. ALT: alanine aminotransferase.

The animals were randomly assigned to five groups (*n* = 4 to 10/group): (1) control group (CON), which received a standard diet commercial feed (Nuvilab, AIN-93-G) and water ad libitum; (2) the high-fat group (HF), which received a high-fat diet (containing fat actioned 20.0%) and water ad libitum (Table 1); (3) the Murici group (Mu-HF), which received murici fruit-based beverages and a high-fat diet; (4) the Tapereba group (Tap-HF), which received tapereba fruit-based bever-ages and a high-fat diet; (5) the blended fruits Murici and Tapereba group (MT-HF), which received murici and tapereba blend fruit-based beverages and a high-fat diet (Figure 1).

The murici and tapereba fruit-based beverages have been developed considering a sensory acceptance experiment conducted with humans and subsequent analysis of antioxidant activity. Following this, murici beverages consisting of 40.0% pulp and 10.0% sucrose and tapereba beverages containing 38.5% pulp and 12.5% sucrose were selected to be given to the animals. The main chemical composition of the drinks of beverages was previously described [13]. Furthermore, a pre-test with murici and tapereba blends was performed and a fruit blend containing 15.0% murici pulp, 7.44% tapereba pulp, and 10.0% sucrose was selected. The fruit-based beverages were offered in bottles and the quantity ingested is shown in Table 2.

The body weight (BW) of animals was measured at the beginning and end of the experimental protocol, and its value is presented as BW gain (i.e., the difference between final and initial BW).

### 2.2. Blood and Tissue Collection

The estrous cycle phase was determined, and animals in estrus were deprived of food for 6 h. Following this, the rats were deeply anesthetized with an intraperitoneal injection of ketamine (100 mg/kg) and xylazine (12 mg/kg) [14].

Blood was collected via cardiac puncture. Samples were separated into two tubes: one with sodium heparin and the other with 0.7% EDTA with 4-(2-aminoethyl) benzenesulfonyl fluoride hydrochloride (AEBSF) 1 mg/mL of blood from Sigma-Aldrich, USA. The samples were centrifuged for 20 min at 314 rad·s^−1^ and stored at −80 °C for analysis.

Livers were collected and weighed to determine the relative weight of the organ denominated by the hepatosomatic index, calculated according to the following formula:

(liver weight (g)/body weight (g)) × 100(1)

### 2.3. Serum Analyses

Capillary blood samples were collected for glucose measurement using the handheld Johnson & Johnson OneTouch^®^ Ultra Mini™ meter. Analysis of the total cholesterol total, high-density lipoprotein (HDL), triglycerides, urea, creatinine, total proteins, albumin, calcium, magnesium, phosphorus, aspartate aminotransferase (AST), and alanine aminotransferase (ALT) was performed using a colorimetric enzymatic assay kit and measured using an automated spectrophotometer (Bioclin System). Inflammatory markers (IL-1β), and tumor necrosis factor-α (TNF-α)], and progesterone levels were quantified using an Enzyme-linked Immunosorbent Assay (ELISA, DuoSet kit, R&D Systems, Minneapolis, MN, USA) according to the manufacturer’s instructions.

### 2.4. Liver Cytokine Analyses

Hepatic levels of TNF-α and IL-1β were detected by enzyme-linked immunosorbent assay (ELISA, DuoSet kit, R&D Systems, Minneapolis, MN, USA) according to the manufacturer’s instructions. Liver samples were collected from rats and homogenized using Triton-X (0.05%) and phosphate-buffered saline (PBS) solution. Samples were centrifuged at 1500× *g* for 10 min and the total protein concentration in supernatants was determined using a BCA Protein Assay Kit (Thermo Scientific, Waltham, MA, USA). The cytokine level measurements (pg/mL) were normalized to total protein level content (pg/mg total protein).

### 2.5. Antioxidant Activity in Plasma and Liver Tissue

#### 2.5.1. DPPH Assay

The radical method used to calculate the antioxidant potential was adapted [15] to 2,2-diphenyl-1-picrylhydrazyl (DPPH), which is based on the quantification of free-radical-scavenging activities. Stock DPPH solution (100 μL; 0.1 μM) was diluted with ethanol (900 μL) and read at 517 nm for absorbance. The sample plasma (100 μL) was added to 900 μL of the DPPH solution for 30 min in the dark, and the decrease in the absorbance was measured at 517 nm using a spectrophotometer (Turner 340, Barnstead/Thermolyne, Dubuque, IA, USA). The samples were subjected to triplicate analysis. The results are expressed as a percentage of radical scavenging.

#### 2.5.2. ORAC Assay

An ORAC assay was applied to the plasma and liver tissue of the animals, employing a 96-well plate reader (SpectraMax i3x, Molecular Devices, San Jose, CA, USA) [7]. Fluorescein was used as a substratum. The fluorescence readings were measured at 485 nm excitation and 520 nm emission. Peroxyl radical was produced using dihydrochloride 2,2’-azobis (2-amidino-propane), which was freshly prepared for each sprint. The experiments were performed at 37 °C in phosphate buffer at pH 7.4.

### 2.6. Apoptosis Assay

Livers were removed and washed with saline solution, following which dissociation of the cells was performed using a 2 mg/L collagenase solution. Flow cytometry analysis was performed immediately. Cells were resuspended in 400 µL of binding buffer containing 5 µL of Annexin V fluorescein isothiocyanate and 5 µL Propidium Iodide (Apoptosis Detection Kit II; BD Biosciences, BD Pharmingen, Mountain View, CA, USA) for 15 min at room temperature (25 °C). Annexin V binding was evaluated by flow cytometry (FACSCalibur; BD Biosciences, Mountain View, CA, USA) after the acquisition of 30,000 events. The data were analyzed in CellQuest (Beckton Dickinson and Company, Franklin Lakes, NJ, USA) and FlowJo v10 (Williamson Way Ashland, OR, USA) software.

### 2.7. Statistical Analysis

The Shapiro–Wilk test verified data normality. The data are presented as means ± standard deviations. Differences between the groups were analyzed using the ANOVA on parametric data and the Kruskal–Wallis test for non-parametric data. Correlations were analyzed with Pearson’s correlation coefficient. Statistical significance was set at *p* < 0.05. The software package used for analysis was GraphPad Prism (version 5.04, GraphPad Software, San Diego, CA, USA).

## 3. Results

The five experimental groups’ body weight gain and diet characteristics after 60 days are depicted in Table 2. After 60 days, there were no significant differences in body weight gain among the groups (*p* > 0.05). The groups consuming fruit-based beverages drank less water than the CON and HF groups (CON and HF vs. MU-HF and Tap-HF and MT-HF groups; *p* < 0.05). There were no significant differences in liver weight or hepatic–somatic levels among the groups (*p* > 0.05) (Table 2).

The metabolic characteristics of the five experimental groups after 60 days are depicted in Table 3. Levels of progesterone, triglyceride, HDL cholesterol total, urea, creatinine, albumin, and total protein were similar among the groups (*p* > 0.05) (Table 3). Glycemia was lower in the group that received the fruit blend than that in the CON group (CON vs. MT-HF group; *p* < 0.05). The serum levels of total cholesterol and liver enzyme (ALT) increased in the HF group, though this was not observed in groups that received the fruit-based drinks (HF vs. Mu-HF and Tap-HF and MT-HF group; *p* < 0.05). The Mu-HF group’s total cholesterol levels reduced to levels similar to those of the CON group (CON = Mu-HF group, *p* > 0.05). The serum calcium concentration was higher in the Tap-HF group than that in the others (Tap-HF vs. the other groups; *p* < 0.05). Additionally, serum magnesium and phosphorus concentrations were reduced in the MT-HF and Mu-HF groups, respectively (*p* < 0.05).

**Figure 2:**
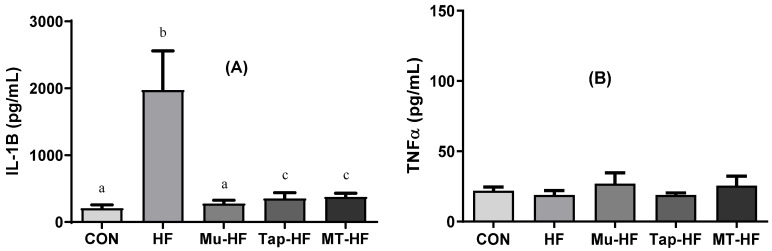
Inflammatory markers in plasma of rats with high-fat diet supplemented with murici and tapereba fruit-based beverages. (**A**) Interleukin-1 beta (IL-1β). (**B**) Tumor necrosis factor-α (TNF-α). CON = control group; HF = high-fat group; Mu-HF = murici beverage group; Tap-HF = tapereba beverage group; MT-HF = murici and tapereba blend group. All values are expressed as mean ± SD. *n* = 4 to 10 per group. Means with different letters are significantly different (*p* < 0.05, Tukey’s test).

**Figure 3:**
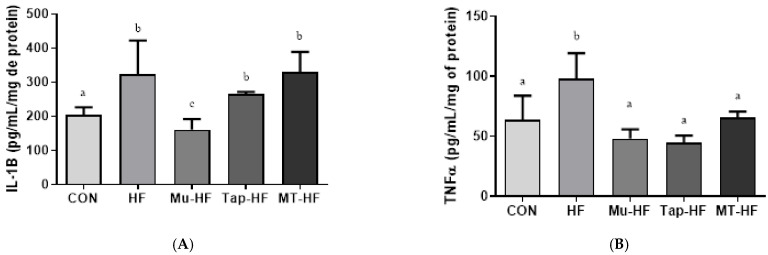
Inflammatory markers in liver tissue of rats with high-fat diet supplemented with murici and tapereba fruit-based beverages. (**A**) Interleukin-1 beta (IL-1β). (**B**) Tumor necrosis factor-α (TNF-α). CON = control group; HF = high-fat group; Mu-HF = murici beverage group; Tap-HF = tapereba beverage group; MT-HF = murici and tapereba blend group. All values are expressed as mean ± SD. *n* = 4 to 10 per group. Means with different letters are significantly different (*p* < 0.05, Tukey’s test).

Plasma and tissue IL-1β levels were higher in the HF group (HF vs. other groups; *p* < 0.05; Figure 2A and Figure 3A). Whilst all fruit-based drinks were effective at reducing IL-1β levels in plasma, consuming murici beverage reduced IL-1β levels in both plasma and the liver (Figure 2A and Figure 3A). There was no difference in the TNF-α plasma levels between the groups (*p* > 0.05; Figure 2B). Compared to the other groups, the HF group had an increased TNF-α level in the liver tissue (HF vs. other groups; *p* < 0.05; Figure 3B); however, consumption of fruit-based drinks prevented this increase.

**Figure 4:**
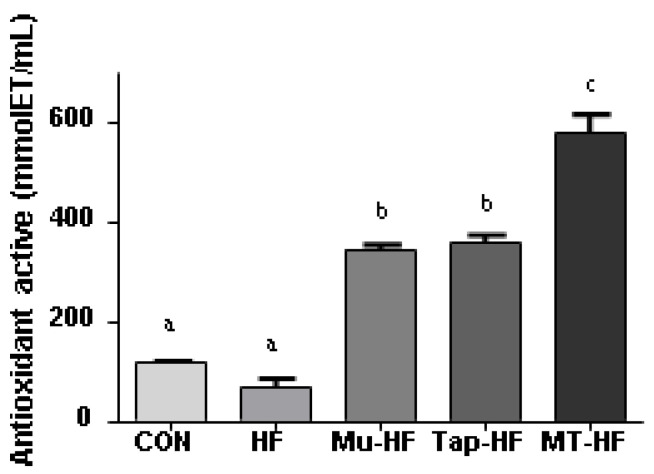
Antioxidant potential for ORAC assay of rat liver tissue cells with high-fat diet supplemented with murici and tapereba fruit-based beverages. CON = control group; HF = high-fat group; Mu-HF = murici beverage group; Tap-HF = tapereba beverage group; MT-HF = murici and tapereba blend group. All values are expressed as mean ± SD. *n* = 4 to 10 per group. Means with different letters are significantly different (*p* < 0.05, Tukey’s test).

Antioxidant activity in the plasma and liver tissue was higher in the groups that received murici and tapereba fruit drinks compared to that in the CON and HF groups (CON and HF vs. other groups; *p* < 0.05; Table 4 and Figure 4). MT-HF showed higher cellular antioxidant activity than the other groups analyzed for ORAC assay (MT-HF vs. other groups; *p* < 0.05; Figure 4).

**Figure 5:**
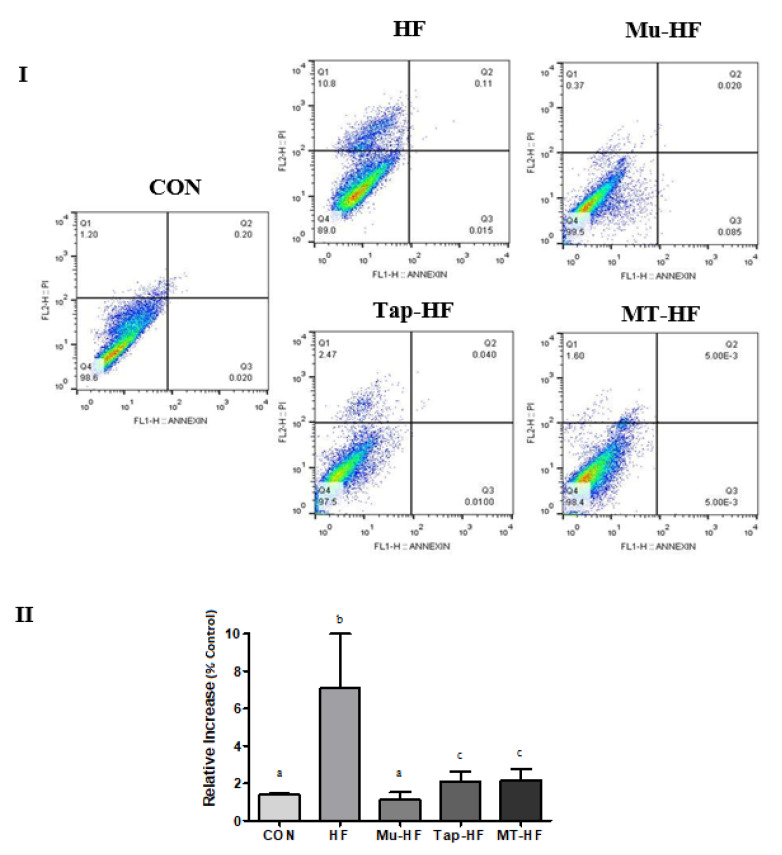
Monitoring of cell death in hepatic cells from rats fed a high-fat diet supplemented with murici and tapereba fruit-based beverages. (**I**) Flow cytometry analysis of hepatic cells from rats fed a high-fat diet supplemented with murici and tapereba fruit-based beverages. (**II**) Quantitative effects of murici and tapereba fruit-based beverages on cell death in hepatic cells from rats fed a high-fat diet. CON = control group; HF = high-fat group; Mu-HF = murici beverage group; Tap-HF = tapereba beverage group; MT-HF = murici and tapereba blend group. All values are expressed as mean ± SD. *n* = 4 to 10 per group. Means with different letters are significantly different (*p* < 0.05, Tukey’s test).

Apoptotic cells were observed in the liver cells of groups HF, Tap-HF, and MT-HF compared to the CON group, except for the Mu-HF group, which showed similar values to the CON group (CON and Mu-HF vs. HF, Tap-HF and MT-HF group; *p* < 0.05; Figure 5). Compared to the CON group, a high-fat diet promoted a decrease in the number of viable cells and a 7.55-fold increase in liver cells (Figure 5). The MT-HF group reversed the increase in apoptosis caused by the high-fat diet, with higher values in the Tap-HF and MT-HF groups compared to those in the CON group (*p* < 0.05; Figure 5).

## 4. Discussion

The Amazon region’s fruits are important sources of bioactive compounds. Product development, such as the preparation of beverages, is a crucial way to increase their consumption [2]. The search for natural products to prevent and treat diseases is still ongoing. Thus, the research interest in the foods’ functional potential increases because they also have positive physiological and metabolic effects on health and nutrition [4].

Fruit pulps have functional compounds, such as vitamins A and C, which have antioxidant activity; carotenoids, such as lutein and zeaxanthin in murici and alpha and beta carotenes in tapereba. In addition to this, polyphenols such as gallotannins, quinic acid gallates, proanthrocyanidenes, quercetin derivatives, galloyl derivatives [2], and fibers control the absorption of nutrients, making them effective adjuvants in the treatment of diseases [6]. No studies similar to this one were found in the literature; thus, studies with other types of foods or experimental designs were used.

In the present study, the animals’ weight gain (g), water consumption (mL), and feed consumption (g) were adequate for the biological moment studied and presented similar results among the groups. This can be explained by the fact that animals can regulate their intake based on the amount of energy consumed [16].

Although there was no statistical difference between the groups’ liver weight and hepatosomatic index, the propensity to increase weight and the hepatosomatic index in Tap-HF suggest a possible organ overload. This finding can be explained by the high beta-carotene concentration of the tapereba fruit in question. Blood glucose in MT-HF was lower than the other groups tested, which can be explained by the lower sucrose concentration and fruit pulp content used in the formulation; therefore, lower fructose content may have contributed to this relationship.

The experimental groups showed no statistical difference concerning their plasma progesterone concentrations in the present study. A similar study performed on female adult Wistar albino rats fed a high-cholesterol diet (standard diet containing 3% cholesterol) demonstrated that hypercholesterolemic experimental groups supplemented with barley grain (10%) or/and date-palm fruit (10%) for 4 months showed a slight improvement in ovarian development, with proliferation and maturation of healthy follicular ovaries [17]. This suggests that, together with a high-fat diet, the administration of these products decreased lipid peroxidation and oxidative stress, leading to normalization of hormonal production.

The intake of fruits and their phytochemical elements (especially polyphenols and fibers) is frequently associated with their ability to reduce or control cholesterol and triglyceride levels in the blood. The effect of polyphenols (particularly flavonoids) on lipid metabolism has not yet been fully elucidated, but a diet rich in bioactive compounds has been found to lead to an increase in plasma HDL and a reduction in LDL [18].

In the current study, it was observed in the Mu-HF group that the dyslipidemia was reversed, and total cholesterol values close to CON were presented. This may be related to the ascorbic acid content of the pulp (58.88 mg/100 g, according to [13]), as well as the presence of other antioxidant compounds acting as protective agents against lipid peroxidation [19]. Consumption of fruit drinks did not affect triglyceride and HDL concentrations in the studied species. Owing to the duration of treatment, no changes in the lipid profile were detected. Storniolo et al. [20] conducted a study using compounds found in sofrito (a tomato-based food and source of carotenoids), and they observed a reduction in LDL oxidation, as well as in oxidative stress in macrophage cell cultures, suggesting that the bioactive compounds from these food sources have beneficial actions.

Creatine and urea are commonly used as serum biomarkers for the glomerular filtration rate [21]. For creatine, no significant difference was observed after ingestion of fruit-based beverages, suggesting that the fruits, together with a high-fat diet, did not cause nephrotic damage to the animals in the concentrations they were given.

Murici and tapereba fruits are sources of phosphorus (7.69 mg/100 g and 26.40 mg/100 g, respectively) [22]. In the present study, a significant difference was observed in the phosphorus levels between the groups that consumed fruit-based beverages and the CON and HF groups. This can be explained by the ingestion of fruits in these beverages, with greater phosphorus in the tapereba based beverage compared to the murici beverage. The mineral magnesium was evaluated and showed higher values in the group that received the murici beverage compared to the groups that received the taperaba beverage or a blend. This variation is because the murici fruit has higher amounts of magnesium in its composition (43.70 mg/100 g) than tapereba (13.55 mg/100 g).

The hepatic function assessment was carried out by assessing the expression of the enzymes ALT and AST, allowing changes in hepatocyte permeability to be determined [23]. Given the data obtained in the study, it is evident that consumption of fruit-based drinks may interfere with ALT and AST values, with consumption reversing the increase in ALT levels until it reached values equal to or less than the CON group. However, a similar study with male Wistar albino rats supplemented with 10% lyophilized mango pulp in a controlled diet for 25 days showed a propensity to decrease ALT and AST values, despite not having a pro-oxidant effect on the liver. [24]. Research with emulsified carotenoids did not alter the serum levels of ALT and AST, demonstrating the opposite of the findings of this study, and this indicates a more powerful effect for the food matrix [25].

A diet rich in fats may activate pathways increasing stress factors, leading to the production of pro-inflammatory cytokines such as IL-1β and TNF-α. The intake of fruit-based beverages in the present study reversed the rise in plasma levels of IL-1β and TNF-α in the liver tissue caused by the high-fat diet. A study by our group showed that supplementing rats with lycopene and tomato sauce demonstrated potential anti-inflammatory action, with their intake showing a propensity towards lowering IL-1β levels. This may be due to the carotenoid content present [26]. These aforementioned pro-inflammatory cytokines regulate adipocyte proliferation and apoptosis [9].

Consumption of the murici and tapereba fruit-based drinks (which are sources of carotenoid) increases the plasma and cell antioxidant capacity of the studied rats’ liver tissue. This indicates an in vivo increase in the defense status of these animals [27], which can be a favorable response to neutralize the production of free radicals. In their study, Storniolo et al. [28] suggest that sofrito bioactive compounds (such as lycopene and beta carotene) can modulate cellular redox status, antioxidant activity cascade, cell growth, and apoptosis in intestinal epithelial cells. Their results showed that lycopene (10 μM) and β-carotene (10 μM) inhibited DNA synthesis around 20% after 48 h of exposure and increased the percentage of cells in the G0/G1 phase in the human cell line of colorectal adenocarcinoma (Caco-2).

In a previous study, our group showed that murici and tapereba fruits are sources of carotenoids (β-carotene and lutein) and phenolic compounds, which grants high antioxidant activity to these fruits [29]. The fruit blend showed a better antioxidant response in the animals, suggesting that the consumption of different fruits together is nutritionally relevant. The increased antioxidant potential in animal tissues and plasma helps to decrease free radicals, offering protection against the noxious effects of reactive species [18].

A potential limitation of this study was that the high-fat diet did not induce important hepatic changes that are characteristic of experimental non-alcoholic liver disease. Future studies can include a high-fat-diet group with animals with symptoms of non-alcoholic liver disease and can also explore other tests for parameters of oxidative stress.

In the present study, it was found that the groups receiving fruit-based drinks also displayed decreased apoptosis levels and a decrease in pro-inflammatory cytokines compared to the HF group. This was similar to the CON group, suggesting a defensive role of bioactive compounds found in fruits in the organs of the animals. Consumption of a hyperlipidic diet results in activation of the gluconeogenesis pathway, resulting in fatty acid oxidation and oxidative phosphorylation for adenosine triphosphate output. Deregulation of this pathway can lead to uncontrolled reactive oxygen species production, which can damage cells, leading to apoptosis [30,31].

When comparing the study groups, it was observed that the MT-HF group presented better results, which can be explained by the composition of the murici fruit, in which carotenoid lutein is found, as demonstrated in previous studies [13,21]. The Tap-HF group also led to improvement in the lipid profile and liver protection compared to HF, but it was inferior to the other groups. The tapereba fruit contains beta carotene, which, as demonstrated in previous studies, can lead to toxicity in the body and liver overload at higher doses [20,26].

## 5. Conclusions

Drinks based on murici and tapereba fruits are important sources of antioxidant compounds that prevent chronic diseases when administered together with a high-fat diet. Their beneficial effects include reducing oxidative stress, lipid oxidation, and the development of pro-inflammatory cytokines, such as IL-1β. A diet’s metabolic effects on the liver are critical to unveil the mechanism of liver damage associated with a high-fat diet.

## Figures and Tables

**Table 1:** Composition of the high-fat diet (20% lipid) prepared at the Experimental Nutrition Laboratory, Department of Nutrition, at the Fluminense Federal University, compared to the formulation proposed by the American Institute of Nutrition for adequate feeding of rats in the growth, pregnancy, and lactation phase (AIN-93).

Components (g/Kg)	AIN-93 G	High-Fat Diet
Casein	200.00	200.00
Starch	397.49	397.49
Dextrin	132.00	132.00
Sucrose	100.00	100.00
L-cystine	3.00	3.00
Soy oil	70.00	70.00
Hydrogenated fat	0	200.00
Mixture of salts (AIN-93G)	35.00	35.00
Vitamin Blend (AIN-93G)	10.00	10.00
Microcellulose	50.00	50.00
Choline	2.500	2.500

**Table 2:** Control of body weight, liver weight, diet intake in experimental groups.

Parameters	CON	HF	Mu-HF	Tap-HF	MT-HF
Initial body weight (g)	181.20 ± 9.63	180.30 ± 10.05	182.30 ± 9.42	183.00 ± 6.45	180.80 ± 10.06
Weight gain (g)	76.20 ± 6.30	88.50 ± 8.42	87.50 ± 15.28	80.80 ± 15.79	79.75 ± 12.69
Feed consumption/group(g)	1040.54 ± 90.59	978.00 ± 49.72	851.87 ± 46.32 *	868.50 ± 123.82 *	811.75 ± 87.63 *
Dietary energy intake (Kcal/Week)	4105.71 ± 356.89	5621.54 ± 286.40	4896.58 ± 266.28	4992.14 ± 711.77	4665.94 ± 503.75
Water consumption/group (mL)	2240.00 ± 330.50	2448.60 ± 423.66	1705.00 ± 525.22 *	1784.00 ± 139.92 *	1788.25 ± 302.81 *
Beverage consumption/group (mL)	−	−	761.08 ± 48.13 *	893.34 ± 41.58	997.27 ± 43.95
Liver weight (g)	7.54 ± 1.00	7.70 ± 1.08	7.64 ± 1.18	8.16 ± 1.02	6.87 ± 0.80
Hepatosomatic index	2.93 ± 0.95	2.87 ± 0.42	2.83 ± 0.39	3.10 ± 0.45	2.63 ± 0.18

CON = control group; HF = high-fat group; Mu-HF = murici beverage group; Tap-HF = tapereba beverage group; MT-HF = murici and tapereba blend group. All values are expressed as mean ± SD. *n* = 4 to 10 per group. * *p* < 0.05 vs. CON group; * *p* < 0.05 vs. HF group.

**Table 3:** Metabolic variable in rats fed with experimental diets.

Parameters	CON	HF	Mu-HF	Tap-HF	MT-HF
Glycemia (mg/dL)	111.4 ± 4.04	109.20 ± 15.09	109.50 ± 23.44	114.55 ± 5.50	97.00 ± 7.87 *
Progesterone (ng/mL)	2.58 ± 0.34	2.51 ± 0.35	2.26 ± 0.75	2.49 ± 0.37	2.34 ± 0.28
Total cholesterol (mg/dL)	46.67 ± 5.69	66.00 ± 6.68 *	50.75 ± 9.84	58.00 ± 5.24 *	57.00 ± 3.80 *
Triglycerides (mg/dL)	59.40 ± 15.34	36.0 ± 10.98	54.80 ± 11.14	48.60 ± 10.31	55.60 ± 16.30
HDL (mg/dL)	23.40 ± 3.58	25.60 ± 3.50	23.60 ± 5.03	26.80 ± 3.11	25.20 ± 1.48
AST (UK)	177.50 ± 30.84	163.00 ± 3.0	174.60 ± 39.64	182.00 ± 35.74	168.00 ± 29.0
ALT (U/mL)	37.60 ± 4.22	42.00 ± 7.34 *	30.75 ± 3.30	39.50 ± 2.64 **	34.75 ± 3.20 **
Urea (mg/dL)	36.50 ± 5.26	31.80 ± 4.86	29.20 ± 2.58	31.60 ± 3.28	34.00 ± 3.16
Creatinine (mg/dL)	0.40 ± 0.08	0.52 ± 0.04	0.46 ± 0.05	0.48 ± 0.08	0.54 ± 0.05
Calcium (mg/dl)	7.64 ± 0.45	7.77 ± 0.18	7.70 ± 0.47	8.90 ± 0.90 *	7.60 ± 0.55
Magnesium	1.12 ± 0.18	0.94 ± 0.19	0.94 ± 0.18	0.84 ± 0.11	0.68 ± 0.13
Phosphorus (mg/dL)	4.26 ± 0.62	4.02 ± 0.65	3.60 ± 0.31 *	4.44 ± 0.71 **	4.74 ± 0.35 **
Albumin (g/dL)	2.42 ± 0.16	2.64 ± 0.19	2.78 ± 0.11	2.85 ± 0.35	2.42 ± 0.13
Protein (µg/L)	5.10 ± 0.36	5.08 ± 0.24	5.42 ± 0.24	5.44 ± 0.57	5.02 ± 0.22

HDL = high-density lipoprotein; AST = aspartate aminotransferase; ALT = alanine aminotransferase; CON = control group; HF = high-fat group; Mu-HF = murici beverage group; Tap-HF = tapereba beverage group; MT-HF = murici and tapereba blend group. All values are expressed as mean ± SD. *n* = 4 to 10 per group. * *p* < 0.05 vs. CON group; ** *p* < 0.05 vs. CON and HF group.

**Table 4:** Antioxidant potential of rats’ serum with high-fat diet supplemented with fruit-based beverages murici and tapereba evaluated by different assays.

Parameters	CON	HF	Mu-HF	Tap-HF	MT-HF
DPPH assay (% reduction)	34.76 ± 5.00	33.52 ± 8.92	51.38 ± 7.94 *	51.85 ± 12.81 *	50.92 ± 2.39 *
ORAC assay (μM TE/g)	196.64 ± 95.91	151.23 ± 37.35	166.15 ± 13.08	461.14 ± 96.07 *	815.34 ± 125.09 **

TE = trolox equivalent; CON = control group; HF = high-fat group; Mu-HF = beverage murici group; Tap-HF = beverage tapereba group; MT-HF = blend murici and tapereba group. All values are expressed as mean ± SD. *n* = 4 to 10 per group. * *p* < 0.05 vs. CON group; ** *p* < 0.05 vs. Tap-HF group.

## Data Availability

All experimental data are shown in the article.
